# Redo aortic root surgery post-Ross procedure

**DOI:** 10.3389/fcvm.2023.1306445

**Published:** 2023-12-12

**Authors:** Alexander Bogachev-Prokophiev, Ravil Sharifulin, Igor Demin, Anastasiia Karadzha, Sergey Zheleznev, Alexander Karaskov, Alexander Afanasyev, Alexey Pivkin, Mikhail Ovcharov, Anton Zalesov, Ivan Murashov, Bashir Tsaroev, Alexander Chernyavsky

**Affiliations:** ^1^ Heart Valve Surgery Department, E. Meshalkin National Medical Research Centre, Novosibirsk, Russia; ^2^ Department of Pathology, E. Meshalkin National Medical Research Center, Novosibirsk, Russia

**Keywords:** Ross procedure, pulmonary autograft, redo surgery, valve-sparing procedure, Bentall procedure

## Abstract

**Background:**

Despite numerous advantages of the Ross procedure, it presents a risk of late autograft and right ventricular outflow tract conduit failure. This study aimed to analyze the outcomes of autograft dysfunction reoperations using autograft-sparing and root replacement techniques.

**Methods:**

Between 2015 and 2023, 49 patients underwent redo root surgery in our institution. Autograft valve-sparing procedures (VSP) were performed in 20 cases and the Bentall procedure (BP) in 29 patients. The short and long-term clinical outcomes along with echocardiographic results of VSP and BP were investigated.

**Results:**

Overall early mortality rate was 2.0% with no significant difference between the groups. Severe autograft valve insufficiency at the time of redo (OR 4.07, *P* = 0.03) and patient age (OR 1.07, *P* = 0.04) were associated with a valve replacement procedure instead of VSP. The median follow-up duration was 34 months. No late deaths occurred in either group. Freedom from VSP failure and aortic prosthesis dysfunction were 93.8% and 94.1% in the VSP and BP groups, respectively. No reoperations were necessary in either group.

**Conclusion:**

Redo aortic root surgery can be safely performed in patients with autograft failure. Both root replacement and autograft valve-sparing procedures demonstrated acceptable results at mid-term follow-up. Early redo surgery pre-empting severe aortic insufficiency increases the likelihood of preservation of the dilated autograft valve.

## 1. Introduction

The Ross procedure yields excellent survival rates, anticoagulant avoidance, and reduced thromboembolism risk (1, 2). However, its main drawback is the risk of redo surgery from autograft and right ventricular outflow tract (RVOT) conduit dysfunction. In various Ross procedures in adults using the freestanding root replacement technique, freedom from autograft reoperation at 15 years was in the range of 75%–94% (3). According to a systematic review and microsimulation by Etnel et al. (4), almost all pediatric patients are projected to undergo autograft redo surgery during their lifetime, whereas in the adult population, this lifetime risk is between 32% and 68%, depending on the age when the Ross procedure was done. Autograft dilatation is the most common cause of late autograft failure, with two options for redo surgery: the Bentall procedure (BP) or valve-sparing procedures (VSP). This study aims to analyze the outcomes of reoperations for autograft dysfunction employing autograft-sparing and root replacement techniques.

## 2. Materials and methods

### 2.1. Study design

This study was approved by the institutional review board (approval number: 2020–58); approval date: October 11, 2020). Informed consent was waived as this was a retrospective analysis of anonymized data. Between December 1998 and May 2023, 1,357 patients (mean age 40.8 ± 17.9 years; range, 7 days to 67 years) underwent the Ross procedures at our center. Consecutive patients, who underwent repeat surgery for autograft failure, were reviewed for this study. Those with indications for BP and VSP aortic root replacement were enrolled. Patients with isolated autograft valve replacement (AVR) or repair were excluded from analysis.

### 2.2. Outcome measures

The primary endpoint was early mortality rate. Secondary endpoints included rates of survival, thromboembolic and hemorrhagic events, major adverse cardiovascular events (death, myocardial infarction, stroke, hospitalization for heart failure), freedom from aortic reintervention, freedom from autograft valve failure/aortic prosthesis dysfunction, and left ventricle/aorta gradients. Postoperative events were evaluated in accordance with the guidelines of the Society of Thoracic Surgeons/American Association for Thoracic Surgery/European Association for Cardio-Thoracic Surgery (5). Early mortality was defined as death from any cause within 30 days of surgery. Late mortality was defined as death 30 days post-surgery. Autograft valve failure was defined as aortic insufficiency grade ≥2. Aortic prosthesis dysfunction was defined as aortic insufficiency grade ≥2, mean transprosthetic gradient ≥40 mmHg, or abnormal prosthetic valve motion. Reoperation was defined as any surgical procedure performed on the root or ascending aorta.

### 2.3. Operative technique

All surgeries were performed through full sternotomy. Extracorporeal circulation was established by cannulation of the distal ascending aorta, right atrium for isolated aortic root surgery, and bicaval cannulation, in case RVOT graft intervention was required. For additional aortic arch procedures, moderate hypothermic circulatory arrest (28°C) with bilateral antegrade brain perfusion was performed. Myocardial protection was achieved using antegrade cardioplegia.

**Figure 1:**
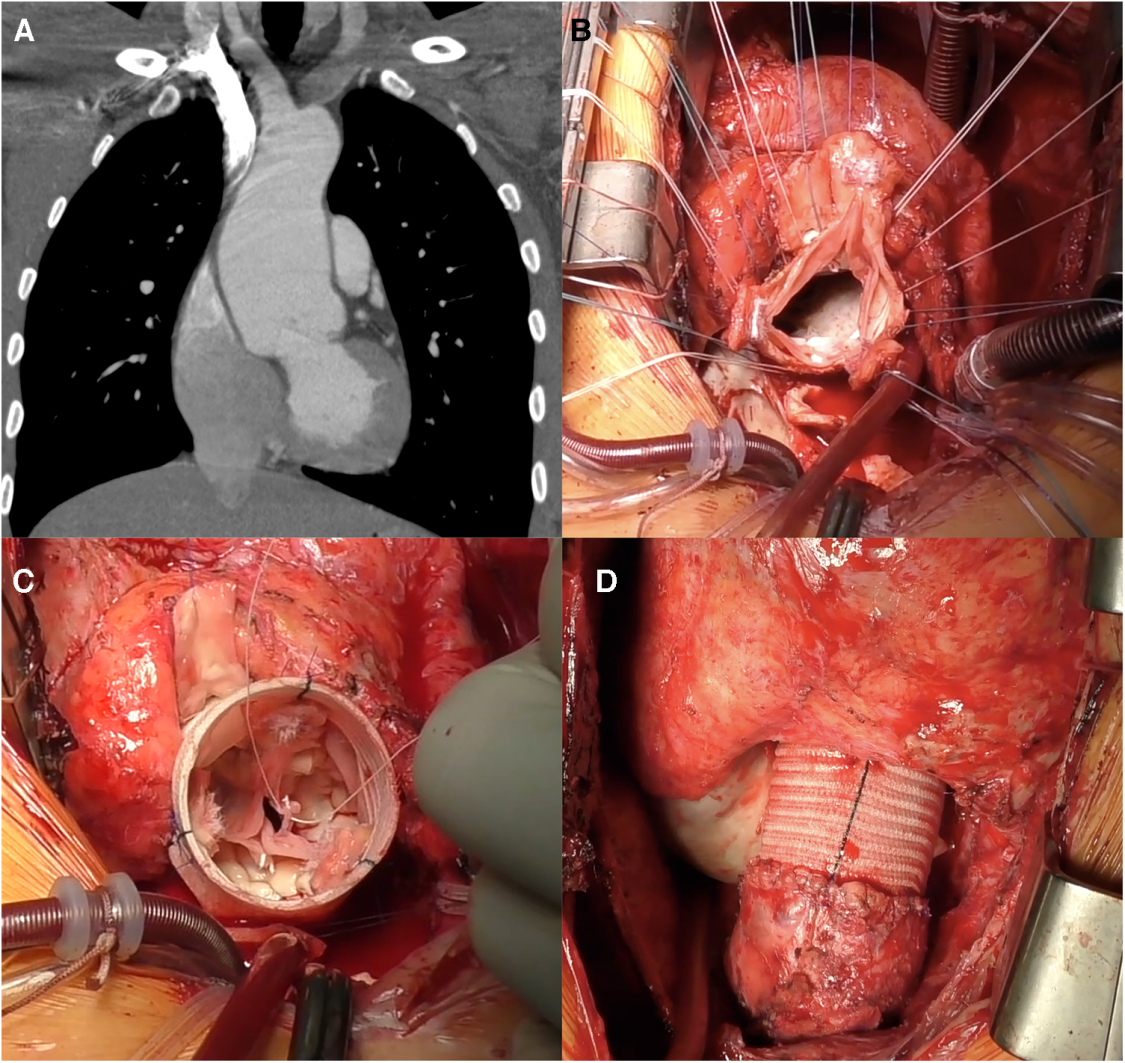
Reimplantation (David) procedure for autograft aneurysm and moderate aortic insufficiency 11 years post-Ross procedure. (**A**) Computed tomography showing neo-aortic root and ascending aorta dilatation up to 56 mm. (**B**) Autograft valve after dissection and resection of the Valsalva sinuses, subvalvular U sutures are placed. (**C**) Aspect of autograft valve after re-implantation inside a tubular Dacron graft and central free margin plication of the leaflets. (**D**) Final view of operation, in the right ventricular outflow tract a new homograft was implanted.

**Figure 2:**
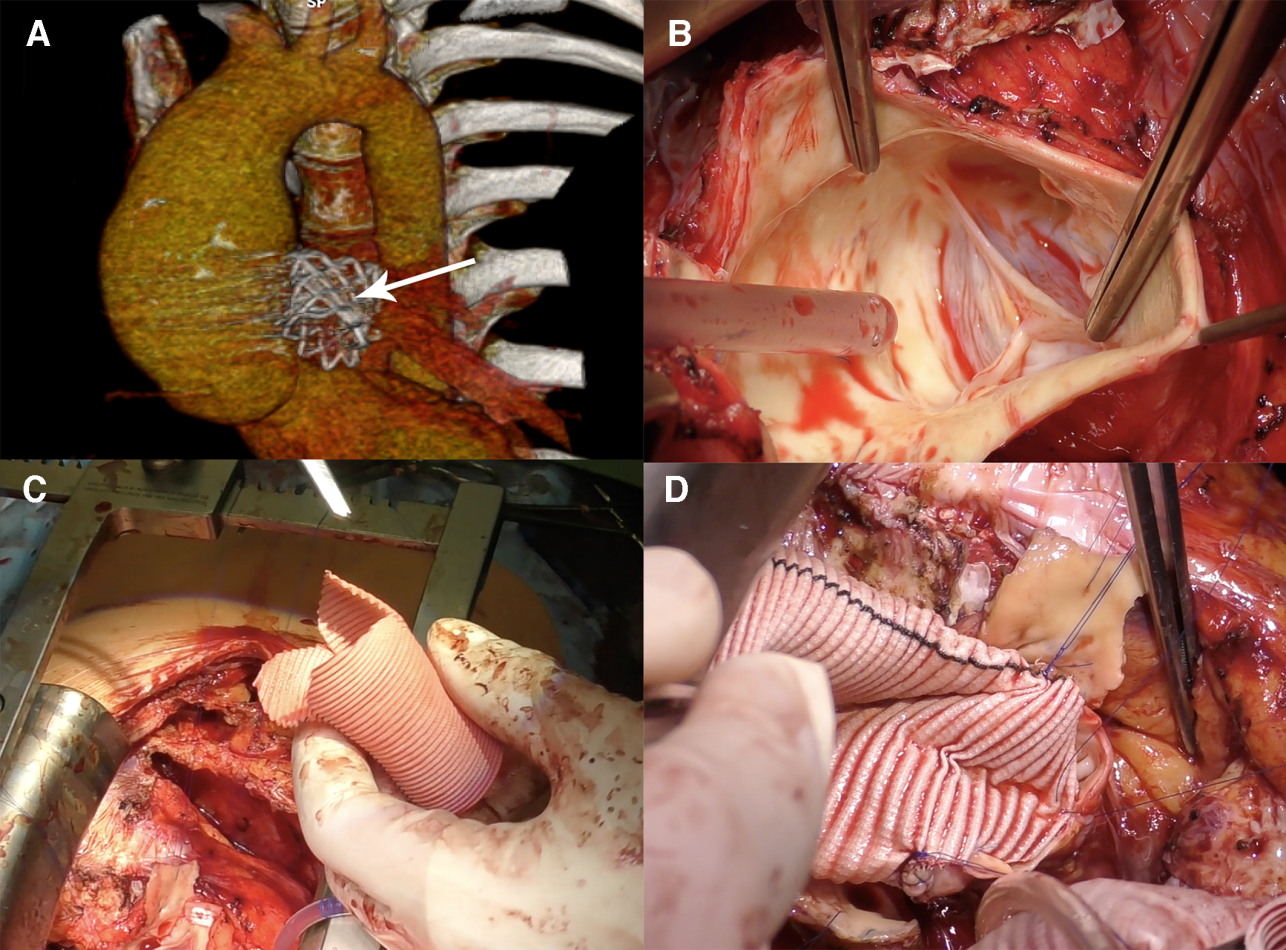
Remodeling (Yacoub) procedure for autograft aneurysm and mild aortic insufficiency in 15 years post-Ross procedure. (**A**) Computed tomography 3D reconstruction showing neo-aortic root and ascending aorta dilatation up to 50 mm, the Melody valve in the pulmonary artery position (arrow). (**B**) Intraoperative view of the pulmonary autograft valve, no degenerative leaflets changes, no annulus dilatation. (**C**) Graft preparation, three tongues are formed. (**D**) The graft is sutured to the aortic root.

The autograft valve-sparing reconstruction techniques were comparable to methods for native aortic valve repair. Reimplantation procedure was commonly used (Figure 1). Autograft sinuses were resected, leaving a 5 mm rim of autograft wall. Deep autograft root dissection down to the basal ring was then performed, as described by El Khoury et al. (6). In redo surgery after a failed Ross procedure, we used a prosthesis with a larger diameter, considering a slightly larger autograft annulus and greater thickness of extra tissue inserted into the prosthesis. Proximal anastomosis was achieved with horizontal U-stitches placed circumferentially throughout the left ventricular outflow tract. The valve was reimplanted into the prosthesis using a continuous suture line with symmetrical commissure orientation at an angle of 120°. Autograft cusp prolapse was addressed using central free margin plication. Lastly, the coronary buttons were re-implanted into the vascular prosthesis. The remodeling technique was used in patients with non-dilated aortoventricular junction (Figure 2).

**Figure 3:**
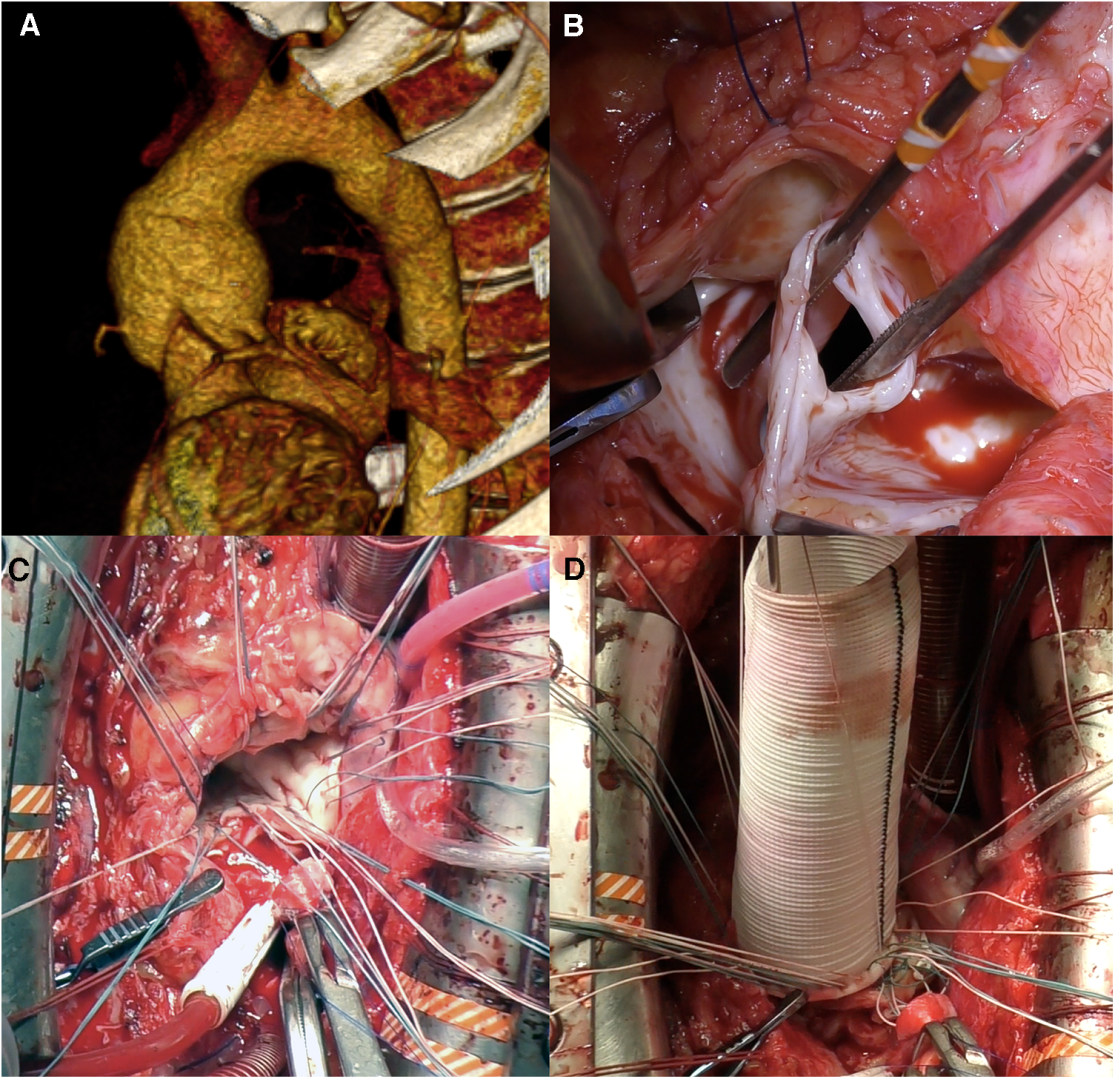
Neo-aortic root replacement (Bentall procedure) for autograft aneurysm and sever aortic insufficiency in 12 years post-Ross procedure. (**A**) Computed tomography showing neo-aortic root and ascending aorta dilatation up to 50 mm. (**B**) Intraoperative view of the autograft valve: fenestrations of noncoronary cusp. (**C**) *U*-shaped sutures are placed circumferentially. (**D**) Neo-aortic root replaced by valved graft.

Root replacement was performed in cases of calcification, restriction, and significant cusp-fenestration. All BP were performed using the button technique with a Cardiamed valved graft [CardiaMed, Penza, Russia, Figure 3] or self-assembled composite biological conduit (stented bioprosthetic aortic valves [NeoCor, Kemerovo, Russia] and vascular graft [Vascutek Ltd, Renfrewshire, Scotland]) fashioned through the “French Cuff” technique (7).

### 2.4. Postoperative drug management

Life-long warfarin anticoagulation therapy was prescribed after AVR with mechanical prostheses, with a median target international normalized ratio (INR) of 2.5. In biological prosthesis implantation, warfarin was prescribed for three months then discontinued in the absence of atrial arrhythmia episodes, based on 24 hour Holter monitoring. In valve-sparing procedures, low-dose aspirin was prescribed for three months, barring other indications for anticoagulants.

### 2.5. Patient evaluation

All patients submitted to preoperative computed tomography angiography to assess aortic diameters and degree of heart adherence to the sternum. Transesophageal echocardiography was performed after bypass weaning to assess the autograft valve or aortic prosthesis function. Transthoracic echocardiography was performed for all patients before hospital discharge. After discharge, examinations including transthoracic echocardiography were scheduled annually. Transvalvular aortic gradients were measured using continuous-wave Doppler ultrasound, while severity of aortic regurgitation was evaluated using color flow Doppler according to the guidelines of the European Association of Echocardiography (8). Aortic regurgitation was graded as none/trivial (0), mild (1), moderate (2), or severe (3). Surgical specimens of the autograft wall and cusps were subjected to a histological examination using hematoxylin and eosin, or Van Gieson's staining. Each period between the time of surgery and the event or end of the follow-up period constituted a separate observation. If the patient was lost to follow-up, we defined the date of the last communication as the censoring date. The follow-up period ran until August 2023.

### 2.6. Statistical analysis

Statistical analysis was performed using STATA, version 14.0 [StataCorp, College Station, TX, USA]. Continuous data are presented as mean ± standard deviation or median (25th and 75th percentiles). Categorical data are presented as counts and percentages. Continuous variables were compared using the independent samples *t*-test for normal distributions or the Mann-Whitney *U*-test for non-normal distributions. Categorical variables were defined using Pearson's chi-square test with an (*N*-1)/N correction factor. The Kaplan–Meier method was used to evaluate survival, with 95% confidence intervals (CIs). Survival curves were compared using the log-rank test. Longitudinal mixed-effects linear regression was used to assess transaortic gradients in dynamics. Logistic regression was applied to identify predictors for performing the BP instead of VSP. Cox regression was used to validate the risk factors for autograft valve repair failure or aortic valve prosthesis dysfunction. Statistical significance was set at *P *< 0.05.

## 3. Results

**Figure 4:**
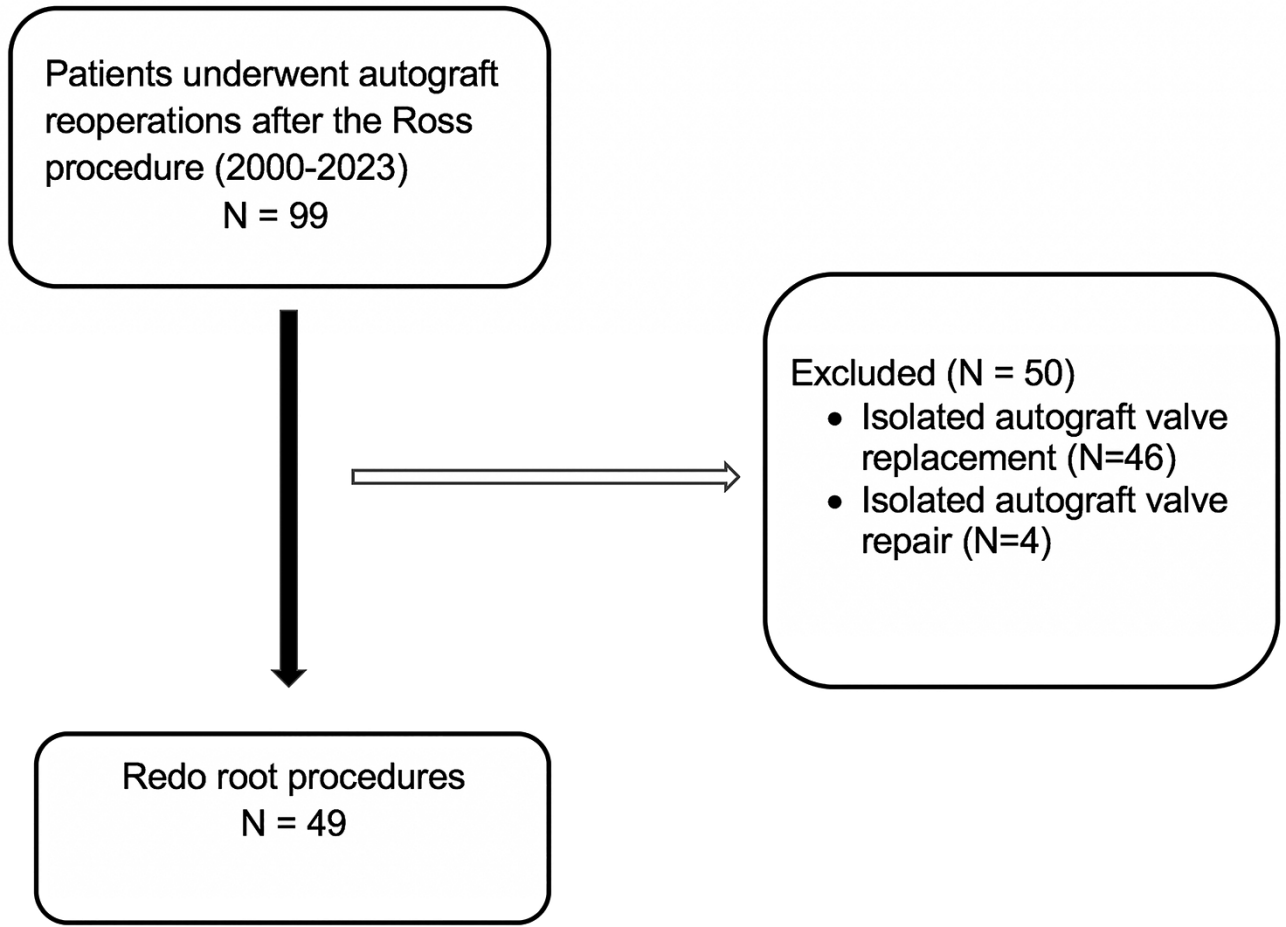
Flow chart of patient enrolment.

**Figure 5:**
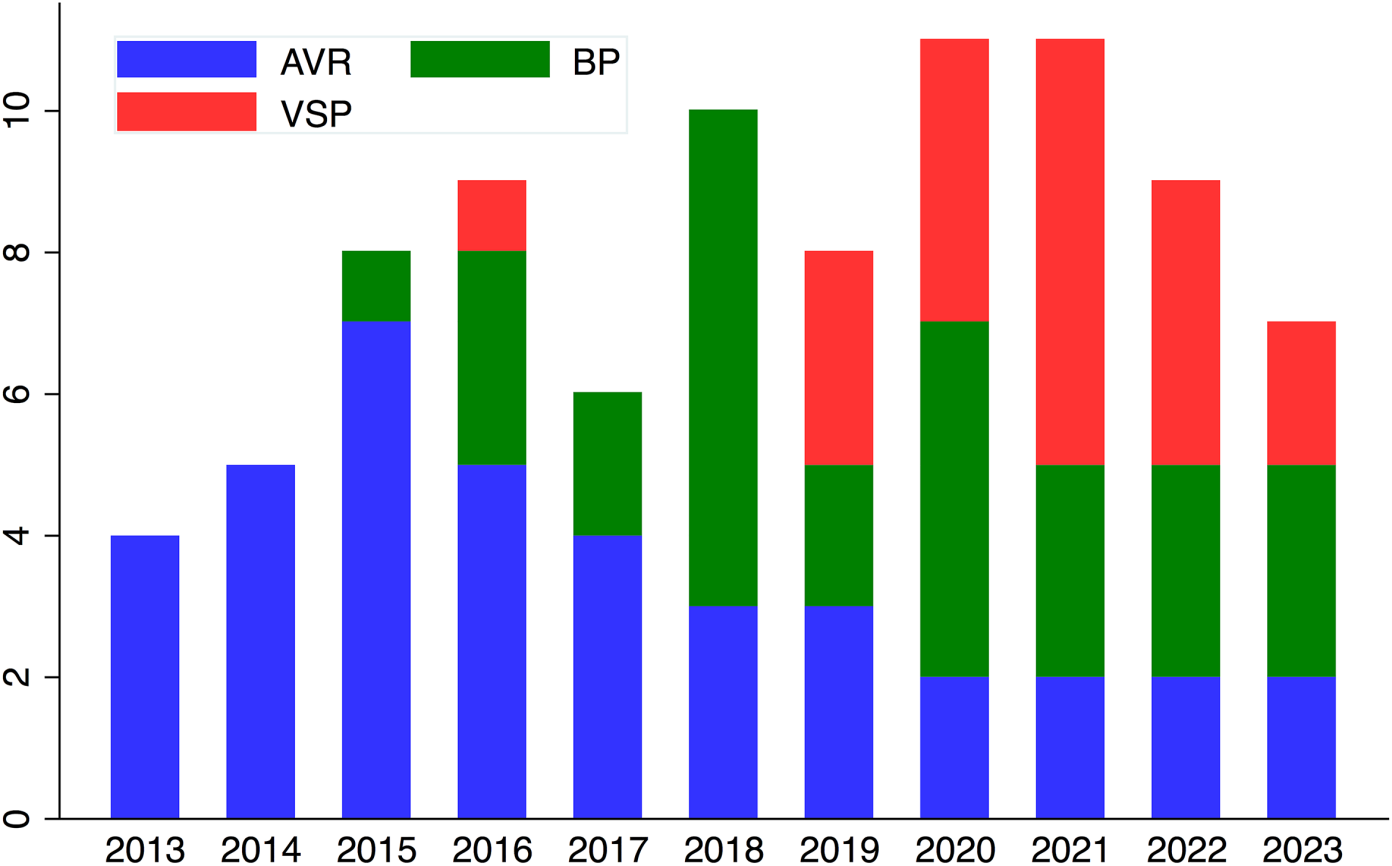
Distribution of procedures over the years. BP, Bentall procedure; VSP, Valve-sparing procedures; AVR, isolated autograft valve replacement or repair.

Forty-nine patients who underwent repeat autograft surgery between January 2015 and May 2023 met the study inclusion criteria (Figures 4, 5). All patients had previously undergone the Ross procedure using the total root replacement technique. Reoperation was performed at a median of 8.7 years post-Ross procedure in patients with a median age of 43.7 years. Patients who underwent VSP were younger than those who did not (*P *= 0.001). A substantial proportion had a bicuspid aortic valve (75.5%), and more than half had isolated aortic regurgitation (53.1%) (Table 1). Most had aortic root dilatation (79.6%), and only a few underwent annulus reduction (10.2%) without autograft reinforcement during the Ross procedure. The main indication for repeat surgery was autograft dilatation with aortic regurgitation (87.8%). There were no differences between the BP and VSP groups in terms of autograft root diameter; the BP group frequently had severe aortic regurgitation grade at the time of redo surgery (*P* = 0.01). Other preoperative patient characteristics are summarized in Table 1.

**Table 1:** Baseline patient characteristics.

	Total	BP	VSP	*P*-value
	(*N* = 49)	(*N* = 29)	(*N* = 20)	
Age at Ross procedure (years)	35.9 (24.0–49.0)	43.5 (35.0–53.0)	18.0 (12.0–30.0)	**0**.**001**
Age at redo (years)	43.7 (30.0–58.0)	49.9 (40.0–59.5)	27.5 (25.0–40.0)	**0**.**001**
Time after Ross procedure (years)	8.7 (5.0–12.0)	7.9 (5.0–11.5)	10.5 (4.4–14.0)	0.25
Body mass index (kg/m^2^)	27.2 (23.9–29.9)	27.9 (26.1–29.9)	24.8 (22.1–29.8)	0.07
Sex (male)	40 (81.6)	25 (86.2)	15 (75.0)	0.32
NYHA class				
NYHA I	1 (2.0)	0	1 (5.0)	0.41
NYHA II	24 (49.0)	14 (48.3)	10 (50.0)	0.91
NYHA III	24 (49.0)	15 (51.7)	9 (45.0)	0.65
Initial aortic valve disease etiology (bicuspid)	37 (75.5)	21 (72.4)	16 (80.0)	0.55
Initial aortic valve hemodynamics				
Insufficiency	26 (53.1)	14 (48.3)	12 (60.0)	0.42
Mixed lesion	13 (26.5)	7 (24.1)	5 (25.0)	0.95
Aortic annulus dilatation (≥27 mm) before Ross procedure	36 (73.5)	21 (72.4)	15 (75.9)	0.75
Annulus reduction during Ross procedure	5 (10.2)	3 (10.3)	2 (10.0)	0.97
Aortic dilatation (>40 mm) before Ross procedure	39 (79.6)	23 (79.3)	16 (80.0)	0.95
Autograft diameter (mm)				
Annulus	29.3 (25.3–32.5)	29.8 (26.0–33.0)	27.7 (24.2–31.5)	0.23
Sinus	47.0 (43.0–50.0)	46.0 (44.0–48.5)	45.5 (41.0–51.5)	0.97
Ascending aorta	47.0 (41.0–51.0)	45.0 (41.0–52.0)	45.0 (41.6–51.0)	0.91
Autograft regurgitation grade				
None/trace/mild	7 (14.3)	2 (6.9)	5 (25.0)	0.08
Moderate	12 (24.5)	5 (17.2)	7 (35.0)	0.16
Severe	30 (61.2)	22 (75.9)	8 (40.0)	**0**.**01**
Autograft valve mean gradient (mm Hg)	4.5 (3.0–5.0)	5.2 (3.0–7.0)	3.0 (3.0–4.0)	**0**.**001**
Indication for redo				
Autograft valve insufficiency + autograft dilatation	43 (87.8)	27 (93.1)	16 (80.0)	0.17
Noe-aorta aneurysm (without valve insufficiency)	4 (8.2)	0	4 (20.0)	**0**.**01**
Subvalvular false aneurysm	2 (4.1)	2 (6.9)	0	0.51
Reasons of autograft failure				
Autograft dilatation	47 (95.9)	27 (93.1)	20 (100)	0.51
Endocarditis	2 (4.1)	2 (6.9)	0	
RVOT conduit function				
No disfunction	22 (44.9)	16 (55.2)	6 (30.0)	0.09
Severe stenosis	12 (24.5)	7 (24.1)	5 (25.0)	0.95
Moderate stenosis	13 (26.5)	6 (20.7)	7 (35.0)	0.27
Moderate insufficiency	2 (4.1)	0	2 (10.0)	0.16

Values are presented as median (interquartile range) and *n* (%). The bold values are *P* < 0.05.

BP, Bentall procedure; NYHA, New York Heart Association; RVOT, right ventricular outflow tract; VSP, valve-sparing procedure.

### 3.1. Intraoperative results

Twenty patients underwent autograft VSPs (40.8%). In the VSP group, the David procedure was the most frequently used (80.0%). There was no intraoperative conversion to AVR for early VSP failure. In 26 (53.1%) cases, pulmonary artery replacement was performed simultaneously because of RVOT graft dysfunction and was required more often in the VSP group (Table 2).

**Table 2:** Operative data and early postoperative outcomes.

Operative data	BP (*N* = 29)	VSP (*N* = 20)	*P*-value
Cusp abnormalities	26 (89.7)	17 (85.0)	0.63
Prolapse only	9 (31.0)	16 (80.0)	**0**.**001**
Retraction	14 (48.3)	0	**0**.**001**
Fenestration	6 (20.7)	2 (10.0)	0.32
Calcification	4 (13.8)	0	0.09
Type of re-operation			
Mechanical Bentall	23 (79.3)	–	
Biological Bentall	6 (20.7)	–	
David procedure	–	16 (80.0)	
Florida Sleeve	–	2 (10.0)	
Yacoub procedure	–	2 (10.0)	
Aortic valve prosthesis size (mm)	24.9 ± 1.7	–	
Graft diameter			
28 mm		4 (20.0)	
30 mm		8 (40.0)	
32 mm		7 (35.0)	
34 mm		1 (5.0)	
Autograft valve repair	–	17 (85.0)	
Central plication	–	16 (80.0)	
Pericardial patch	–	2 (10.0)	
Bypass time (min)	193.5 (171.5–210.0)	220.0 (194.0–234.0)	0.06
Aortic cross-clamp time (min)	130.8 (113.0–150.0)	157.5 (142.0–170.0)	**0**.**002**
Concomitant procedures			
Arch replacement	2 (6.9)	1 (5.0)	0.79
RVOT conduit reoperation	12 (41.4)	14 (70.0)	**0**.**05**
Mitral valve repair	7 (24.1)	0	**0**.**02**
Tricuspid valve surgery	4 (13.8)	1 (5.0)	0.32
Atrial fibrillation ablation	2 (6.9)	1 (5.0)	0.79
Coronary artery bypass grafting	1 (3.4)	0	>0.99
Early mortality	1 (3.4)	0	>0.99
Postoperative ventilation time (hours)	17.0 (5.0–30.0)	17.0 (12.0–39.0)	0.35
Intensive care unit duration (days)	3.0 (2.0–5.0)	3.0 (2.0–4.0)	0.72
Re-exploration for bleeding	3 (10.3)	1 (5.0)	0.51
Myocardial Infarction	1 (3.4)	0	>0.99
ECMO	1 (3.4)	0	>0.99
Atrial fibrillation paroxysms	9 (31.0)	5 (25.0)	0.65
Permanent pacemaker	1 (3.4)	0	>0.99
Stroke	1 (3.4)	1 (5.0)	>0.99
Wound infection	2 (6.7)	0	0.51

Values are presented as the mean ± standard deviation, median (interquartile range), and *n* (%). The bold values are *P* < 0.05.

BP, Bentall procedure; ECMO, extracorporeal membrane oxygenation; RVOT, right ventricular outflow tract; VSP, valve-sparing procedure.

Multivariate logistic regression analysis identified more severe autograft valve insufficiency at the time of redo [OR 4.07 (95% CI, 1.11–14.89, *P* = 0.03)] and older age [OR 1.07 (95% CI, 1.00–1.14, *P* = 0.04)] as factors associated with performing a valve replacement procedure instead of valve-sparing (Table 3).

**Table 3:** Predictors of autograft valve replacement instead of valve-sparing (logistic regression model).

Risk factor	Univariable model		Multivariable model	
	OR (95% CI)	*P*-value	OR (95% CI)	*P*-value
Sex (men)	0.71 (0.16–3.19)	0.66	–	–
Age at redo surgery	1.09 (1.04–1.16)	**0**.**001**	1.07 (1.00–1.14)	**0.04**
Time post-Ross procedure	0.92 (0.82–1.04)	0.18	0.96 (0.79–1.16)	0.70
Year of the surgery	0.81 (0.60–1.09)	0.16	0.75 (0.48–1.19)	0.23
Aortic regurgitation grade	3.62 (1.53–8.56)	**0**.**003**	4.07 (1.11–14.89)	**0.03**
LV \ aorta mean gradient	1.82 (1.09–3.01)	**0**.**02**	1.76 (0.93–3.34)	0.08
Annulus diameter	2.65 (0.57–12.39)	0.22	–	–
Sinus diameter	0.79 (0.27–2.34)	0.68	–	–
Tubular aorta diameter	1.26 (0.58–2.71)	0.56	–	–

The bold values are *P* < 0.05.

CI, Confidence Interval; LV, Left ventricle; OR, Odds ratio.

### 3.2. Early morbidity and mortality

There was one early death in the BP group (3.4%) and none in the VSP group. The 58-year-old man in the BP group suffered acute myocardial infarction diagnosed in the operating room. Urgent angiography showed left coronary artery ostial stenosis up to 90%. The patient underwent percutaneous coronary intervention with one drug-eluting stent implantation *and good angiographic results.* The patient died 12 days after surgery due to multiorgan failure. The groups did not differ in complication rate (Table 2). One patient in each group had an ischemic stroke. Two patients in the BP group and one patient in the VSP group underwent re-exploration for bleeding.

### 3.3. Late mortality and survival

**Figure 6:**
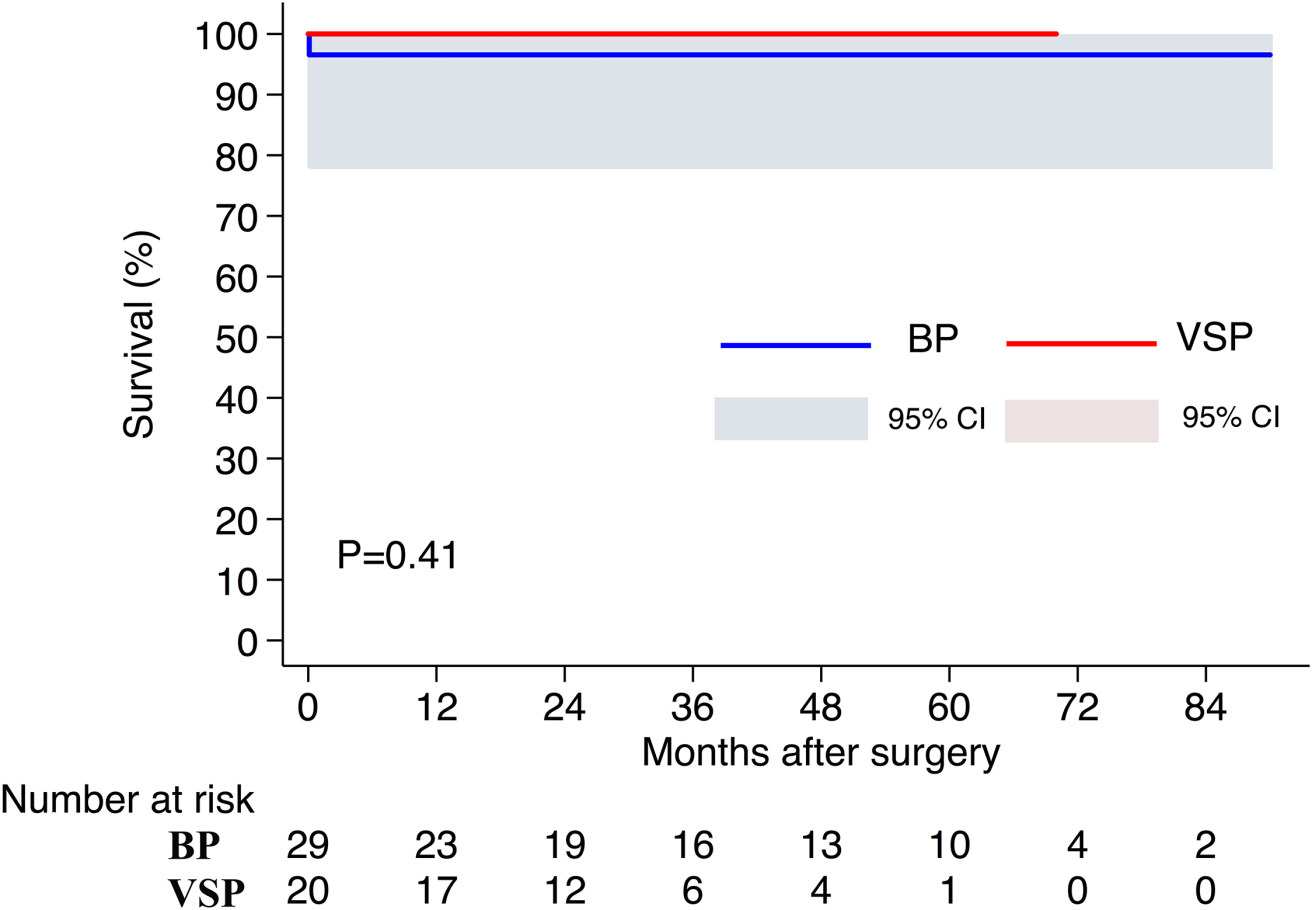
Survival rates. BP, Bentall procedure; VSP, valve-sparing procedure.

Follow-up was longer in the BP group (40.0 [13.0–62.0] vs. 25.0 [18.0–42.5] months, *P* = 0.12). Follow-up data were available for 27 (96.4%) and 20 (100%) patients in the BP and VSP groups, respectively. No late deaths occurred in either group. Survival rates were 96.5% (95% CI, 77.6–99.5) and 100%, respectively (*P* = 0.41, Figure 6). In the latest follow-up, 85.2% of patients (*n* = 23) were in New York Heart Association class I or II in the BP group and 90.0% (*n* = 18) in the VSP group (*P* = 0.63).

### 3.4. Thromboembolic and hemorrhagic events

There was one ischemic stroke in the BP group and no major hemorrhagic events in either group at late follow-up (Table 4). Twenty-three patients (85.1%) in the BP group continued to receive warfarin. In the VSP group, two patients (10.0%) were on oral anticoagulation for atrial fibrillation.

**Table 4:** Late results.

Late results	BP (*N* = 27)	VSP (*N* = 20)	*P*-value
Follow-up, months	40.0 (13.0–62.0)	25.0 (18.0–42.5)	0.12
Late mortality	0	0	–
Survival, % (95% CIs)	96.5 (77.6; 99.5)	100%	0.41
Root Re-operations	0	0	–
Freedom from VSP failure/ aortic prosthesis dysfunction, % (95% CIs)	94.1 (65.0; 99.2)	93.8 (63.2; 99.1)	0.69
Permanent pacemaker	1 (3.7)	0	>0.99
Stroke	1 (3.7)	0	>0.99
Myocardial Infarction	0	0	
Hospitalization for heart failure	0	0	
Major adverse cardiovascular events	1 (3.7)	0	>0.99
Hemorrhagic events	0	0	

Values are presented as median (interquartile range), *n* (%), or % (95% confidence intervals (CIs). BP, Bentall procedure; VSP, valve-sparing procedure.

### 3.5. Echocardiographic results

At discharge, the BP group demonstrated a higher mean LV/aorta gradient than the VSP group (10.0 [9.0–11.0] vs. 5.0 [4.0–5.0] mmHg, *P *< 0.001). At follow-up, the transprosthetic gradient was also significantly higher in the BP group (Table 5). Longitudinal mixed-effects linear modelling revealed significant changes in gradients over time in both groups (0.49 ± 0.11 mm Hg/year, *P *< 0.001 and 0.51 ± 0.12 mm Hg/year, *P *< 0.001 for the BP and VSP groups, respectively). None of the patients had mean gradients exceeding 40 mmHg. One patient had moderate aortic regurgitation in each group (Table 5). There was significant ventricle volume reduction at 1 year without differences between groups (Table 5).

**Table 5:** Echocardiographic data.

Variable	BP	VSP	*P*-value
Baseline	*N* = 29	*N* = 20	
LV ejection fraction at baseline (%)	56.0 (52.0–61.0)	59.0 (57.0–64.0)	0.09
LVEDV index (ml/m²)	86.8 (75.3–115.1)	61.1 (47.9–93.8)	**0**.**02**
LVEDD index (cm/m²)	2.81 (2.65–3.48)	2.54 (2.17–3.08)	**0**.**04**
Discharge	*N* = 28	*N* = 20	
LV ejection fraction at discharge (%)	55.0 (47.0–59.0)	58.0 (55.0–65.0)	0.07
LVEDV index at discharge (ml/m²)	64.8 (53.9–80.2)^a^	52.0 (43.8–67.9)	**0**.**03**
LVEDD index at discharge (cm/m²)	2.46 (2.26–2.74)^a^	2.45 (2.18–2.69)^a^	0.48
Peak LV\aorta gradient at discharge (mm Hg)	19.0 (18.0–21.0)	8.5 (7.0–10.0)	**<0**.**001**
Mean LV\aorta gradient at discharge (mm Hg)	10.0 (9.0–11.0)	5.0 (4.0–5.0)	**<0**.**001**
Aortic regurgitation ≥2 at discharge	1 (3.6)	0	>0.99
Follow-up	*N* = 27	*N* = 20	
Echo follow-up (months)	38.0 (13.0–60.0)	23.0 (17.5–37.0)	0.11
LV ejection fraction at follow-up (%)	55.3 (46.6–64.4)	49.2 (41.6–56.3)	0.94
LVEDV index at 1 year (ml/m²)	55.3 (46.6–64.4)^a^	50.5 (44.3–59.2)^a^	0.12
LVEDD index at 1 year (cm/m²)	2.38 (1.99–2.53)^a^	2.33 (2.14–2.61)^a^	0.65
Peak LV\ aorta gradient at follow-up (mm Hg)	23.0 (18.0–23.0)	11.0 (10.0–15.0)	**<0**.**001**
Mean LV\ aorta gradient at follow-up (mm Hg)	11.0 (10.0–14.0)	7.0 (5.0–7.7)	**0**.**001**
Aortic regurgitation ≥2 at follow-up	1 (3.7)	1 (5.0)	>0.99

Values are presented as median (interquartile range), or *n* (%). The bold values are *P* < 0.05. BP, Bentall procedure; LV, left ventricle; LVEDD, left ventricular end-diastolic diameter; LVEDV, left ventricular end-diastolic volume; VSP, valve-sparing procedure.

^a^ *P*-value < 0.05 in comparison with baseline data.

### 3.6. VSP failure/aortic prosthesis dysfunction and re-interventions

Freedom from VSP failure and aortic prosthesis dysfunction were 93.8% (95% CI, 63.2–99.1) and 94.1% (95% CI, 65.0–99.2), respectively (*P *= 0.69). Multivariate analysis did not identify risk factors associated with failure of autograft valve repair or aortic valve prosthesis. No reoperations were performed in either group.

### 3.7. Histological data

**Figure 7:**
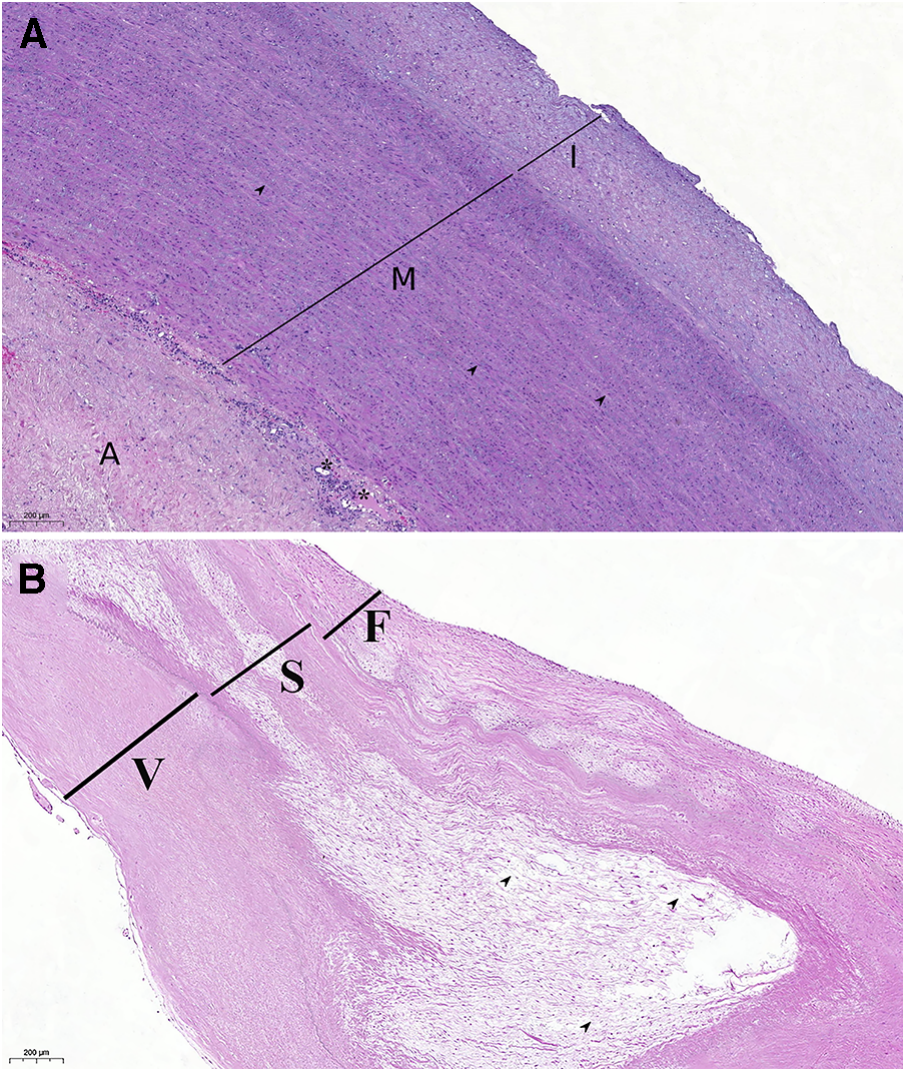
Histological examination of autograft wall and leaflets, hematoxylin, and eosin stain. (**A**) Autograft wall. Intima thickening, disorganization of elastic fibers in the media (arrowheads), neovascularization on the border of media and adventitia (vasa vasorum, asterisk). A: adventitia; I: intima; M: media. (**B**) Pulmonary autograft leaflet. Thickening of the ventricularis (**V**), degenerative change of the spongiosa (**S**), and disorganization of collagen fibers (arrowheads). F: fibrosa.

Histological examination of the autograft wall revealed a preserved, layered structure. The intima and adventitia were significantly thicker. Elastic fibers in the media were unbent and broken. Diffuse sclerotic changes, insignificant inflammatory infiltration, and an increased quantity of vasa vasorum on the media and adventitial borders were observed (Figure 7). Pulmonary autograft leaflet explants showed a conserved three-layered structure with a thickened ventricularis. In some places, degenerative changes in the middle layer were noted. No inflammatory changes or calcium deposition were observed (Figure 7).

## 4. Discussion

This study demonstrates that redo aortic root surgery can be safely performed in patients with autograft failure despite its complexity, even if both autograft and RVOT conduit interventions are necessary.

Previous studies have reported that reoperations post-Ross procedure are associated with high perioperative risks (9). In a recently published series, it was demonstrated that in expert centers, reoperations post-Ross procedure can be performed with low morbidity and mortality, ranging from 0% to 3% (10–12). In the study by Shih E. et al, there was no difference in 15-year survival between patients who underwent redo surgery (58 autograft reinterventions) and those patients who did not require reoperation after the Ross procedure (13).

After the root replacement technique, the main mechanism of autograft failure is progressive dilatation (4), which makes VSPs possible. Majority of patients with failed autografts are still young at the time of reoperation; VSPs conserve the advantages of a functioning autograft valve, including oral anticoagulants avoidance and increased survival. Histological examination in our series revealed that even if valve replacement is necessary, autograft cusps stay alive and maintain architecture resembling that of the native aortic valve without inflammation and calcification, as confirmed by other histological studies (14).

According to the literature, valve-sparing reoperations post-Ross procedure, despite its technical complexity in experienced hands, is a safe option with successful autograft salvage rates ranging from 50% to 90% with excellent survival of 85%–92% at 8–10 years (3, 15, 16).

Both remodeling and reimplantation methods have been successfully applied to failed autografts, with the surgical approach largely depending on the surgeon's preference. The Yacoub technique underscores its benefits during redo surgery, with less need for root dissection and protection of autograft leaflets from injury on the Dacron graft (17, 18). Conversely, Yacoub-type valve-sparing without additional annuloplasty does not allow to achieve durable annulus stabilization. However, currently available data have not demonstrated a significant difference in 10-year durability between reimplantation or remodeling techniques.

In the study by Liebrich et al., the David procedure was performed in 18 patients with autograft dilatation (19). No early mortalities occurred. In a mean follow-up of 3.2 years, one patient underwent AVR; in others, freedom from aortic regurgitation of grade 2 or greater was 100% at three years.Schäfers et al. reported on 18 root remodeling procedures post-Ross (18). The authors suggest managing a dilated annulus by external suture annuloplasty with an expanded polytetrafluoroethylene suture. Freedom from reoperation at 8 years was 88% (mean follow-up of 5.4 years).

In a multicenter European study, 87 valve-sparing procedures were performed post-Ross procedure, including 37 reimplantation and 17 remodeling procedures, with an early mortality rate of 1.2% (15). The authors found that in most patients with autograft dilatation after the root technique, valve preservation could be successfully performed with acceptable mid-term results. On the other hand, after the subcoronary and inclusion cylinder techniques, severe isolated regurgitation without dilatation is more often observed due to degenerative changes in the leaflets. Consequently, prosthetic valve replacement is inevitable in most patients. At eight years, freedom from autograft valve reintervention was 85% in patients with valve-sparing autograft root replacement and only 33% in those with isolated autograft valve repair (15).

Ratschiller et al. (3) reportedexperience of 27 valve-sparing reoperations for autograft aneurysms (10 David; 17 Yacoub procedures), with no early mortality. The mean follow-up duration was 4.6 years. Freedom from autograft reintervention was comparable to that reported in a previous study (86.6% at five years). All three reinterventions were performed after the Yacoub procedure.

**Figure 8:**
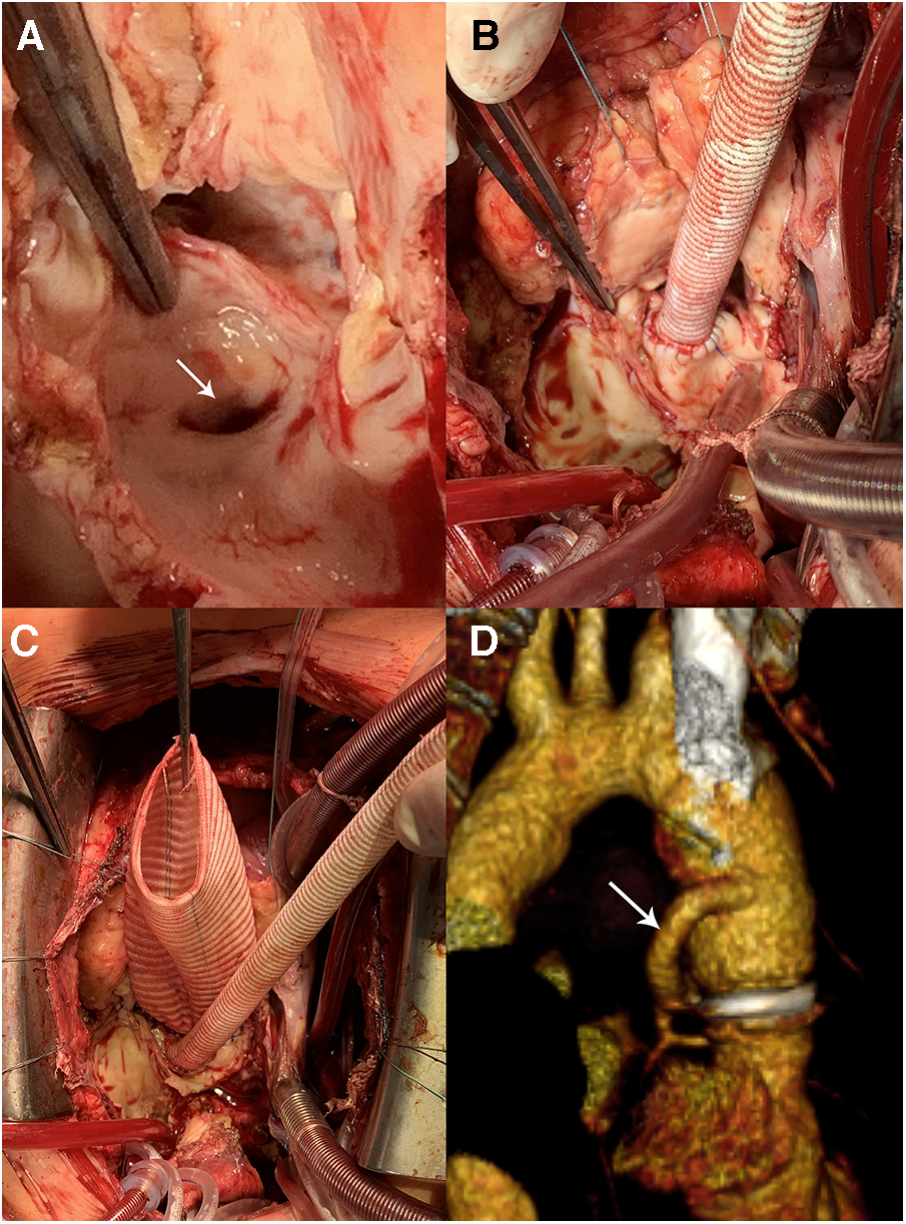
Bentall procedure and hemi-Cabrol graft-left main anastomosis. (**A**) The left main coronary artery ostium (arrow) is close to the autograft annulus. (**B,C**) Нemi-Cabrol graft-left main anastomosis. (**D**) Computed tomography angiography after Bentall procedure and hemi-Cabrol graft-left main anastomosis (arrow).

In a recently published study by Jahanyar et al. (16), which is the largest single center study on valve-sparing root replacements after the Ross procedure (39 reimplantation and 3 remodeling procedures) with the longest follow-up (7.8 years), 10-year survival and freedom from reoperation on the neo-aortic valve were 92.4% and 79.7%, respectively. Thus, available studies on redo root surgery after the Ross procedure (3, 15, 16, 19) enrolled relatively few patients and have limited follow-up (in the range from 3.0 to 7.8 years). However, current data demonstrate 80%–86.6% freedom from reintervention at 8–10 years after autograft valve preservation, which is comparable to the results after native aortic valve repair (15, 16, 19). This suggests that valve-sparing autograft root replacement is a viable and perspective approach. However, larger studies, including meta-analyses, and long-term follow-up data are needed. In our series, the mortality rate was 2.0% in patients who underwent redo root replacement and VSPs, consistent with the literature data. We launched VSPs post-Ross procedure in 2015. These operations require extensive experience in aortic root reconstructive surgery. Deep aortic root dissection is technically challenging, particularly around the autograft root adhesion with the RVOT conduit. Additional difficulties may arise during mobilization of the left coronary artery, especially if replacement of the RVOT conduit is unscheduled, requiring the use of the modified Cabrol technique in a couple of cases (Figure 8). Moreover, autograft commissures are often located at different levels, which requires expertise to correctly reimplant into a vascular prosthesis. We initially preferred the BP because it is less technically demanding (Figure 5); but with the accumulation of significant experience with VSPs in patients with native tricuspid and bicuspid aortic valve insufficiency, we have fundamentally changed our approach. Patient age is one of the factors affecting the choice of redo procedure. Young patients with autograft failure first of all are considered for valve-sparing surgery. We are convinced that we should try to spare the valve if the objective for Ross was to avoid anticoagulation. However, surgeon should not preserve the autograft valve at any cost and must be aware of re-intervention risk. VSPs are performed only in cases where there is no doubt about the durability of the autograft valve. In cases of calcification, restriction, and large fenestrations, requiring additional complex reconstructions on the leaflets, we tend towards valve replacement. The decision to perform autograft VSP should be decided after an informed discussion with the patient regarding the risk of another reoperation. In patients older than 60 years, we give preference to bio-Bentall procedure instead of valve-sparing due to a more predictable result.

Since most patients at the time of redo surgery have aortoventricular junction dilatation, we prefer the reimplantation technique, as it allows effective stabilization of the annulus. The remodeling technique was used in two patients with non-dilated annulus.

In our series, both the BP and VSPs demonstrated comparable results at mid-term follow-up in terms of survival, freedom from thromboembolic, and hemorrhagic events. Significant differences between the groups were found only in the transaortic gradients, which were lower after sparing procedures and did not translate into any clinical benefits. However, the follow-up period of this study was relatively short requiring further analysis.

Currently, there is no consensus on the indications for autograft dysfunction surgery. We believe that surgical tactics should be more active. Our regression analysis showed that with an increasing degree of autograft valve insufficiency at the time of redo surgery, the likelihood of performing valve-sparing surgery decreases (20). We agree with most authors that in the presence of autograft dilatation, postponement of surgery for severe regurgitation leads to degenerative changes in the leaflets and reduces the success rate of VSP (15, 17, 19–21). Thus, early surgery is aimed primarily at preserving the autograft's own living valve and not at preventing aortic events, which are rare. In our center, indications for redo surgery post-Ross procedure are severe aortic insufficiency or an autograft diameter of 5 cm or more, even in the absence of valve dysfunction.

**Figure 9:**
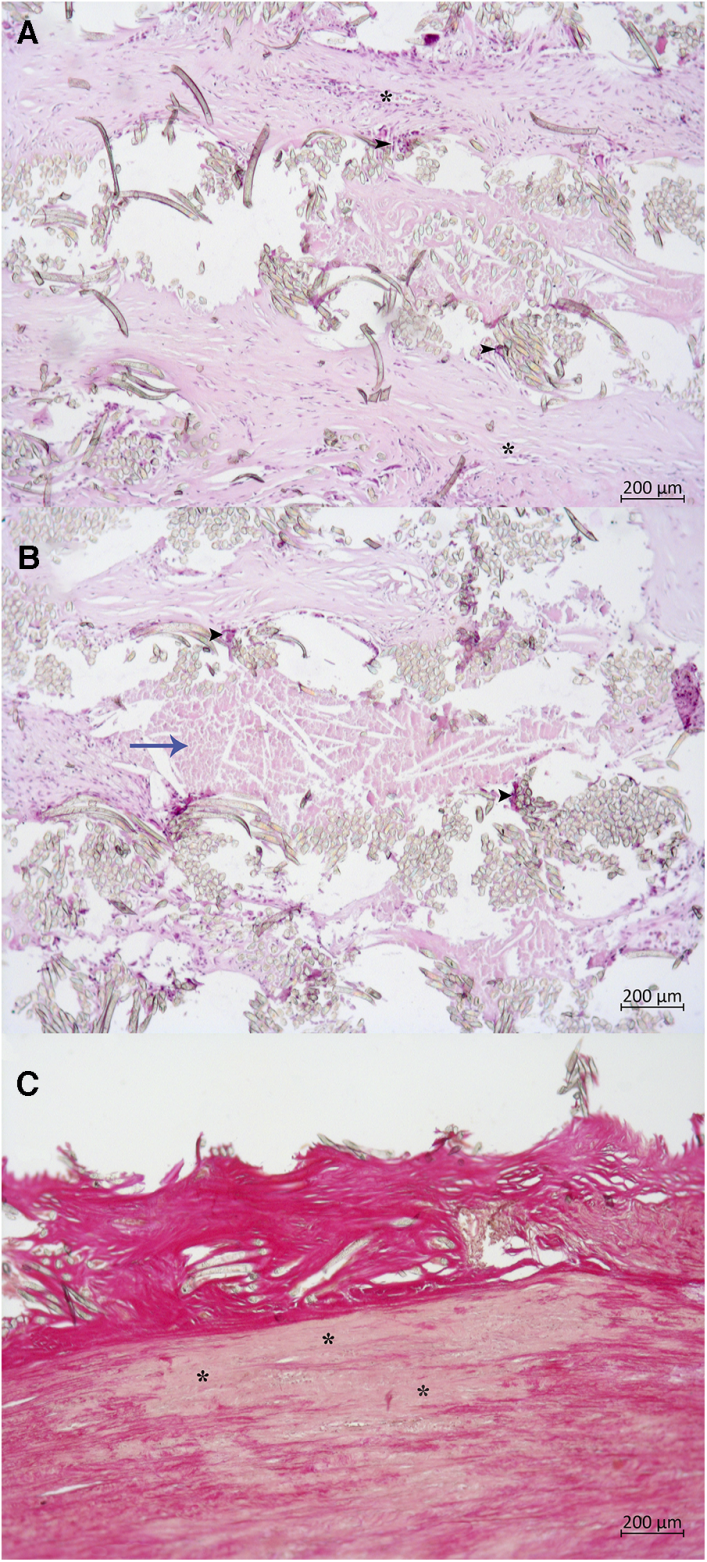
Histological examination of autograft wall wrapped with Dacron graft. (**A,B**) Dacron graft with surrounding cellular reaction, hematoxylin, and eosin: many foreign body giant cells (black arrowheads), lymphocytes, macrophages. Dense fibrosis outside of the fabric (blue arrow). The asterisk (*) marks a neovessel. (**C**) Degenerative changes in the tunica media, Van Gieson's stain: Thinning of media, medionecrosis (asterisk), smooth muscle atrophy and apoptosis, breaking of elastic fibers.

The most typical patient undergoing redo surgery in this series, was the subject with a congenital bicuspid aortic valve with pure insufficiency or mixed aortic valve disease, aortic annulus and root dilatation, without autograft reinforcement from the primary surgery. Recognizing the high incidence of late autograft dilatation, we modified implantation technique and routinely applied external support of the autograft with a Dacron graft in adult patients. Since 2017, the pulmonary autograft inclusion technique has been used in 60 adult patients. Presently, only two patients underwent early reoperation after autograft reinforcement, due to a technical error. In the series by Starnes et al. (22), in one of the largest long-term follow-up studies on autograft reinforcement (58 patients with a median follow-up of 4.3 years), the pulmonary autograft inclusion technique reduced the pulmonary autograft reintervention rate. There were three early reoperations, all within a year post-Ross procedure, possibly due to technical errors, as in our report. In reinforced autograft dysfunction, valve-preserving correction will be futile, since in the absence of dilatation, the culprit will be leaflet degeneration. Moreover, there is concern regarding the condition of the autograft wall once encased in a rigid vascular prosthesis. In the above-mentioned cases, histological examination revealed degenerative changes in the tunica media, including smooth muscle atrophy, apoptosis, elastic membrane fragmentation, and chronic cellular inflammatory reaction around the vascular prosthesis (Figure 9). These findings are consistent with histopathologic data from aortic and pulmonary autografts wrapped with low-porosity grafts in experimental studies (23). Whether this affects the long-term results of the Ross procedure is unclear and requires further study.

In conclusion, despite their complexity, valve-sparing procedures can be safely performed in patients with autograft failure. We believe the most suitable for autograft VSP are young patients with mild or moderate aortic insufficiency, without organic changes of leaflets, and who consciously chose valve repair procedure despite the risk of another re-intervention.

The mid-term results of redo root surgery were acceptable; however, the follow-up duration of the study was short, and a longer follow-up is necessary to assess the durability of valve-sparing techniques for autograft-valve preservation.

## Limitations

This study was a single-center study with a non-randomized design; thus, had several limitations. When analyzing the predictors of autograft replacement, it was unfeasible to consider all factors affecting the choice of treatment method. First, it is a surgical experience, confirmed by the predominance of BPs in the early stages as less technically demanding operations. Moreover, in some cases, patients preferred the BP to minimize the risk of further re-intervention. Additionally, VSPs and BP had slightly different indications. VSP was used in a selective group of patients with less pronounced autograft leaflets changes. The mean follow-up period was relatively short. Further evaluation of the long-term results is necessary.

## Conclusion

Redo-aortic root surgery can be safely performed in patients with autograft failure. Both root replacement and autograft valve-sparing procedures demonstrated acceptable results at mid-term follow-up. Early redo surgery before aortic insufficiency becomes severe increases the likelihood of preservation of the dilated autograft valve.

## Data Availability

The raw data supporting the conclusions of this article will be made available by the authors, without undue reservation.
